# West Nile Virus: A Neglected Cause of Bell’s Palsy?

**DOI:** 10.7759/cureus.62486

**Published:** 2024-06-16

**Authors:** Ibrahim Al-Hashimi, Emmanuel Okon

**Affiliations:** 1 Department of Internal Medicine, University of Texas (UT) at Tyler Health Science Center, UT Health East Texas, Tyler, USA; 2 Department of Infectious Diseases, Christus Trinity Clinic, Tyler, USA

**Keywords:** bell’s palsy, cranial neuropathy, acute encephalitis, mosquito-borne diseases, neuroinvasive west nile virus

## Abstract

The West Nile virus (WNV) is the leading cause of mosquito-borne disease in the United States. Bell’s palsy (BP) is a clinical syndrome associated with viral infections, but an association with West Nile virus (WNV) is not well-described, with only two cases reported in the literature. We present a case of a 68-year-old woman presenting with fevers and encephalopathy. Cerebrospinal fluid was positive for WNV. Following improvement, she developed facial weakness and was diagnosed with BP secondary to the WNV infection. Identifying BP associated with WNV infection may have significant clinical implications, but further studies are needed to fully characterize a causative relationship.

## Introduction

West Nile virus (WNV) is a mosquito-borne flavivirus that was first identified among infected birds in Africa, Asia, and Southern Europe. Introduced to the United States in 1999 via New York City, it is now found across the US, as well as Canada and Mexico, and is the primary cause of mosquito-borne encephalitis. The major vectors in the United States are the *Culex* species of mosquito. About 20% of humans infected develop symptoms, most commonly an acute febrile syndrome, which is often associated with a maculopapular rash [1]. One in 50 people develops a neuroinvasive disease, including encephalitis, meningitis, and acute flaccid paralysis [2]. Other reported clinical syndromes include chorioretinitis, hepatitis, pancreatitis, and myocarditis. Bell’s palsy (BP) is a clinical syndrome characterized by inflammation of the seventh cranial nerve (CN VII), causing unilateral facial paralysis. It can be a significant cause of morbidity, ranging from psychological distress in mild cases to motor and sensory disturbances in severe cases. Several viruses have been implicated in the development of BP, but only two single-case reports in the literature have associated it with WNV [3,4]. We report another case of BP onset shortly following the acute phase of neuroinvasive WNV encephalitis.

## Case presentation

A 68-year-old woman with a past medical history of supraventricular tachycardia, hypertension, hyperlipidemia, prediabetes, and hypothyroidism presented to an emergency department in Texas in late July 2023, complaining of a one-day history of fever, inappropriate speech, headache, and double vision. Her travel history included a two-week vacation trip to Spain in June, returning a month prior to the presentation, during which time she swam in a resort pool but did not report insect bites or ill contacts. The only notable exposure to animals included small scratches or bites from her dog, which was fully vaccinated.

On initial presentation, vital signs showed an oral temperature of 39.2°C (102.6°F), a heart rate of 92, a blood pressure of 121/67, a respiratory rate of 18, and an oxygen saturation of 92% on room air. On a physical examination, she was alert and oriented to the person, time, and place. She followed commands appropriately but had expressive aphasia. Cranial nerve examination showed a decreased right shoulder shrug. Motor examination revealed 4/5 power in the right upper extremity (RUE) with a motor drift. The physical examination was otherwise unremarkable.

A complete blood count was notable for a platelet count of 118 K/dL. Contrast-enhanced computed tomography (CT) and magnetic resonance imaging of the brain were unremarkable. She was started empirically on ceftriaxone 2000 mg intravenously (IV), vancomycin 15 mg/kg IV, ampicillin 2000 mg IV, doxycycline 100 mg IV, and acyclovir 10 mg/kg IV. Cerebrospinal fluid (CSF) analysis demonstrated a white blood cell count of 126 cells/mm3 and a red blood cell count of 3 cells/mm3 with 76% neutrophils and 18% lymphocytes, a protein of 193 mg/dL, and a glucose of 65 mg/dL. Ceftriaxone, ampicillin, and vancomycin were discontinued due to the elevated protein and normal glucose, suggesting a viral etiology. On hospital day four, she became poorly responsive to verbal and tactile stimuli and was intubated and mechanically ventilated for airway protection. CT imaging of the brain without contrast was repeated and showed no abnormalities. Two days later, a CSF enzyme-linked immunosorbent assay (ELISA) showed a positive West Nile virus immunoglobulin M (IgM) at 3.27 international units/milliliter (IV/mL) and a negative WNV IgG at 0.50 IV/mL. CSF polymerase chain reaction (PCR) was negative for herpes simplex virus-1, cytomegalovirus, enterovirus, human herpes virus-6, varicella-zoster virus, parechovirus, and cryptococcus. The serum ELISA was positive for WNV IgM and negative for IgG. CSF was not tested for WNV-ribonucleic acid (RNA), as it was not readily available in our institution. On hospital day eight, her mental status improved, and she was extubated successfully. On hospital day 10, she became unable to close her right eye with the flattening of the right nasolabial fold. She was diagnosed with postinfectious Bell’s palsy secondary to an acute WNV infection and started on prednisone and acyclovir. Acyclovir was discontinued after four doses, given a high clinical suspicion that the WNV infection was the inciting cause. Her mental status and right upper extremity weakness returned to baseline over the next three days, and she was discharged to a rehabilitation facility with planned outpatient follow-up in an infectious diseases clinic.

## Discussion

Bell’s palsy (BP) refers only to idiopathic seventh cranial nerve (CN VII) neuritis [5] and thus excludes, for example, Lyme-associated facial nerve palsy. Although various viral infections have long been suspected to be a cause of BP, in clinical practice, the difficulty and impracticality of confirming viral infections as the pathologic mechanism means that even when suspected, virus-associated cases are labeled as Bell’s palsy. Although viral inflammation has been associated with the development of BP, a growing body of evidence suggests a multifactorial etiology is likely [6]. CN VII has a long and tortuous anatomic pathway that makes it particularly susceptible to palsy: it originates from the facial nucleus in the pons, extends to the internal auditory meatus intracranially, has a bony segment through the fallopian canal, and finally emerges from the stylomastoid foramen and continues into the parotid gland. Anatomical variations amongst patients may contribute to the incidence, severity, and laterality of BP [7,8]. In addition to these anatomic factors, histopathological examination of CN VII in BP shows inflammatory infiltration by small, round, mononuclear cells, which supports an inflammatory or immune cause. The identification of herpes simplex virus type 1 (HSV-1) DNA in endoneurial fluid specimens in 1996 has since been considered evidence of HSV-1 reactivation as one such cause [9]. Other viruses implicated include Epstein-Barr virus, cytomegalovirus, human immunodeficiency virus, influenza virus, and SARS-CoV-2, among others [10-14]. Bacterial infections such as Rickettsia and Ehrlichia have also been associated, which, coupled with the association of BP with vaccination [13], suggests that an immune response may be more responsible than the direct neurotoxicity of infections. This is supported by the time of onset of BP following the initial insult. In our case, BP developed after the patient defervesced and mental status improved, about 10 days following the onset of symptoms. Nonimmune mechanisms may also contribute to the development of BP. Nerve ischemia has been postulated to be a contributor, as BP is more common in patients with diabetes mellitus and hypertension. The increase in incidence of BP in the physiologically fluid-retaining state of pregnancy, like other compression neuropathies, supports edema as a contributor to BP, further evidenced by the association with pre-eclampsia [15]. Campbell and Brundage demonstrated in 2002 that acute cold exposure is a potential trigger of BP, later confirmed by several studies [16].

In our case, we diagnosed a neuroinvasive WNV infection as the trigger. Although our patient had prediabetes and hypertension, both were well controlled. She had positive herpes simplex serum antibodies, but her CSF was negative, and she had no history of active infection. However, these factors may have increased her baseline risk of developing BP. Her presentation in late summer and lack of a travel history to cold areas rule out cold exposure. Flaviviruses are very rarely associated with BP. In addition to the two WNV-associated cases reported, Peter et al. reported in 2013 a case of dengue virus infection presenting with BP [17], and an association with the Usutu virus is also documented [18].

Historic data on WNV infections recorded by the Centers for Disease Control and Prevention from 1999-2022 show a hospitalization rate of 46% and a case fatality rate of 5%, rising to 73% and 9%, respectively, in neuroinvasive disease. Around 95% of deaths were in cases of neuroinvasive disease [19]. Complications from longstanding Bell's palsy, regardless of cause, include facial synkinesis and incomplete eye closure [20].

## Conclusions

WNV is one of the few disease-causing flaviviruses endemic to the United States and is now the leading cause of mosquito-borne disease in the United States. It is associated with significant morbidity and mortality, and neuroinvasive involvement is particularly lethal, with nearly all deaths in WNV infections occurring in neuroinvasive disease. Bell's palsy may also lead to bothersome and potentially serious complications. A better understanding of the epidemiology and clinical presentation of West Nile virus infections, including a possible association with BP, is important, and further studies are needed to fully characterize a causative relationship.
